# Epidemiology of road traffic accidents and its associated factors among public transportation in Africa: systematic review and meta-analysis

**DOI:** 10.3389/fpubh.2025.1511715

**Published:** 2025-02-28

**Authors:** Yibeltal Assefa Atalay, Bersufekad Wubie Alemie, Belete Gelaw, Kelemu Abebe Gelaw

**Affiliations:** ^1^School of Public Health, College of Health Science and Medicine, Wolaita Sodo University, Wolaita Sodo, Ethiopia; ^2^Department of Ophthalmology and Optometry, Hawassa University College of Medicine and Health Science, Hawassa, Ethiopia; ^3^School of Nursing, College of Health Science and Medicine, Wolaita Sodo University, Wolaita Sodo, Ethiopia; ^4^Department of Midwifery, Menelik II Medical and Health Science College, Addis Ababa, Ethiopia

**Keywords:** road traffic accidents, prevalence, associated factors, drivers, systematic review, meta-analysis, Africa

## Abstract

**Introduction:**

Nowadays, Injuries, disabilities, and deaths due to road traffic accidents pose a major threat to public health worldwide. There was no meta-analysis study conducted in this area in Africa, hence the need for the study.

**Objectives:**

This review aimed to assess the pooled prevalence and associated factors of road traffic accidents among public transportation in Africa.

**Methods:**

This systematic review and meta-analysis study was conducted in Africa according to the Preferred Reporting Items for Systematic Reviews and Meta-Analyses (PRISMA) guidelines. Using Boolean logic operators and targeted keywords, we searched for publications on several electronic databases (Web of Science, PubMed, Google Scholar, African Journals Online (AJOL), and Science Direct). The degree of heterogeneity among the included studies, the 95% confidence interval, and the pooled prevalence were estimated using a random effects model.

**Results:**

This review included 45 studies with 15,968 participants. The overall estimated pooled prevalence of road traffic accident among public transportation in Africa was found to be 38.83% (95% CI: 33.54, 44.12). Meta-regression analysis indicated that sample size, publication year, country, study design, and sub-region had no significant impact on RTA prevalence. Based on a sub-group analysis by countries where the studies were conducted, the higher pooled prevalence of road traffic accidents was found in Congo at 67.90% (95% CI: 59.99, 75.81), while the lower pooled prevalence of road traffic accidents was in Libya at 15.80% (95% CI: 10.99, 20.61). Driving experience (AOR: 2.64, 95% CI: 1.70, 3.34), chewing Khat while driving (AOR = 4.69, CI: 2.80, 7.85), alcohol use (AOR = 2.72, CI: 1.49, 4.97), and receiving mobile phone calls while driving (AOR = 2.37, CI: 1.42, 3.95) were factors significantly associated with road traffic accidents.

**Conclusion:**

In conclusion, the pooled prevalence of road traffic accidents in Africa was found to be high. Therefore, we recommend that drivers have to strictly adhere to traffic regulations Moreover, It is recommended that policymakers and administrators ought to gain awareness of road traffic accidents and its risk factors to put existing road traffic accidents preventive and control measures into action.

## 1 Introduction

World Health Organization (WHO) characterizes road traffic accidents as injuries, whether fatal or non-fatal, that arise from collisions occurring on public roadways. These incidents must involve at least one moving vehicle and may also include pedestrians. RTA is defined as any crash that takes place on a street or thoroughfare accessible to public traffic, leading to the death or injury of one or more individuals, with the involvement of at least one moving vehicle (1). Road traffic accidents encompass collisions involving vehicles, interactions between vehicles and pedestrians, encounters between vehicles and animals, as well as incidents involving vehicles and various geographical or architectural barriers. This phenomenon represents a significant yet often overlooked public health issue that necessitates concerted efforts aimed at achieving effective and sustainable preventive measures (1, 2).

An accident is an event that takes place unexpectedly, arising under circumstances that were not anticipated (2). RTA represent significant global public health and developmental challenge, resulting in approximately 1.2 million fatalities annually, and causing injuries or disabilities to between 20 and 50 million individuals worldwide. Consequently, these incidents led to an economic loss estimated at 518 billion US dollars on a global scale (3).

The 2013 World Health Organization report revealed that over 1.24 million people die each year from road traffic injuries, making it the eighth leading cause of death globally and the top cause for those aged 15–29. If trends continue, it is expected to become the fifth leading cause of death by 2030 (4). In spite of their significant effects on global health and economic progress, RTA are often given insufficient priority in low-and middle-income countries (5).

In the developing world, the improved life expectancy together with industrialization and urbanization are putting heavy pressure on the transport system in general and on the road system in particular (6). In addition, when compared to the developed nations, causes of high burden in road traffic-related deaths and injuries in developing countries are primarily due to an increase in motor vehicle numbers, poor enforcement of traffic safety regulations, inadequacy of public health infrastructure, and poor access to health services (7).

In Africa, approximately 2,400 individuals lose their lives daily due to injuries, with road traffic accidents being the predominant cause. The mortality rate from road traffic injuries in African nations is 40% greater than that observed in other low-and middle-income countries, and it exceeds the global average by 50% (8).

Furthermore, there exists a significant disparity globally regarding the utilization of roads and the existence of injuries, which carries substantial implications for formulation, and implementation of road safety policies and practices. In nations with high levels of motorization, road traffic accidents predominantly involve car drivers. Conversely, in specific Asian countries, motorcycle riders are more frequently involved, while in numerous low-income nations, the victims often include occupants of various passenger vehicles, walkers, and factors related to road infrastructure and vehicle design. Additionally, exposure to risk factors such as speeding and the failure to wear seatbelts further exacerbates the situation (9).

Even though the registered vehicle count in Africa remains relatively low, the estimated rate of road traffic fatalities is significantly high. In 2015, the ratio of vehicles per 1,000 individuals in Africa stood at 46.6, in stark contrast to 510.3 in Europe (10). Many African nations lack effective policies to protect vulnerable road users, and data collection on key risk factors like alcohol use, speed regulation, seat belt use, child safety restraints, and helmet compliance is infrequent. In addition, there is also lack of effective enforcement of traffic laws and regulations in the African region (11).

To our knowledge, no comprehensive analysis of road traffic accidents has been conducted in Africa. Our aim was therefore to compile and analyze the available data on the epidemiology of road traffic accidents and its associated factors in Africa. Given that RTA is a widespread public health problem, the results of our study will underscore the importance of a holistic approach to understanding and containing this problem. Consequently, the key findings of this review will have a significant impact on policymakers, researchers, and stakeholders, encouraging them to establish and strengthen collaborative efforts across sectors.

Moreover, this study will serve as basis for development of national and international strategies, protocols, and guidelines. Ultimately, the results of this research will have a significant impact on preventing and controlling road traffic accidents, and used as making a sole input to the literature.

## 2 Methods

### 2.1 Study protocol and registration

The purpose of this review is to determine the pooled prevalence of RTA and their associated factors among public transportation in Africa. The study’s accuracy and comprehensiveness were ensured using the PRISMA 2020 checklist (12) (Supplementary Table S1). The review protocol has been submitted to an international register for systematic reviews to ensure accountability.

### 2.2 Searching strategy

Various databases were used to conduct a comprehensive search for relevant studies, including, PubMed, Cochrane Library, Google Scholar, Science Direct, and African Journals Online. The literature search was limited to studies published in English that examined the pooled prevalence of RTA among drivers of public transportation in Africa. To ensure comprehensive coverage of the literature, the reference lists of the included studies were carefully checked. A systematic approach was followed to conduct an advanced search on PubMed. Initially, search terms were formulated for eight areas: “road traffic accidents,” “prevalence,” “accidents,” “road traffics,” “drivers” “public transportation” “Associated Factors” and “Africa.” These keywords were retrieved from Google Scholar and then individually searched in PubMed to identify relevant MeSH terms within the MeSH hierarchy tree. These terms were then combined using advanced Boolean search logic, specifically using the “AND” and “OR” operators to effectively bring the concepts together. These search terms were selected based on the PECCO-Principles selected to ensure retrieval of relevant articles from the above databases. All searches were limited to papers written in English and the last search in all databases was performed on the 22th July 2024.

### 2.3 Population, exposure, context, condition, and outcomes (PECCO) frameworks

P=Population: drivers. E = Exposure: The level of exposure plays a crucial role in influencing the adherence to road traffic accidents by drivers in Africa. These factors include driving experiences, chawing Khat while driving, alcohol use, and receiving mobile phone calls while driving. C=Context: Africa. C=Condition: public transportation.

O=Outcome measurement: The main objective of the research was to assess the prevalence of road traffic accidents among drivers of public transportation in Africa. Furthermore, the study sought to investigate the predictors of road traffic accident involvement.

### 2.4 Inclusion and exclusion criteria

This study included studies that met specific criteria. These criteria included having a population of drivers, focusing on the prevalence of road traffic accidents and their associated factors. The studies were conducted exclusively in Africa and were published in English. However, certain primary studies were excluded for various reasons. These reasons included a lack of information on the prevalence of road traffic accidents, unavailability of the full text, low-quality score, inability to access the full text after multiple attempts to contact the corresponding author, and Non-primary studies, such as review articles, conference papers, and editorials, were excluded.

### 2.5 Quality assessment

Three authors (YAA, KAG, and BG) independently assessed the standard of the studies using Joanna Briggs Institute (JBI) standardized quality assessment checklist (13). The disagreements raised during the quality assessment were resolved through a discussion led by the third author (BWA). Eventually, the dispute was settled and an agreement was reached. The critical analysis checklist contains eight parameters with the options Yes, No, Unclear, and Not Applicable. Studies were considered low risk when they scored 50% and above on the quality assessment indicators, as reported in a Supplementary Table S2.

### 2.6 Methodological quality assessment

We employed the instruments developed by Hoy et al. (14) to evaluate both internal and external validity based on ten established criteria, aiming to assess the potential risk of bias. The potential biases were categorized as either low (with a total score of 6 to 8), moderate (with a total score of 3 or 5), or high (with a total score of 0 to 2). Ultimately, only articles with minimal risk of biases were included in this comprehensive evaluation (Supplementary Table S3).

### 2.7 Data extraction

Four authors (YAA, KAG, BG, and BWA) working independently abstracted the relevant data from studies by using a standardized Microsoft Excel spreadsheet. The JBI tool for prevalence studies was used as a guideline for data extraction from the finally selected articles. The data extraction tool contains information on the author’s name, year of the study, title, year study was conducted and year of publication, country, sub-region, study design, and type, sample size, and the outcome measured, prevalence rate, Information regarding the publication status, and the study quality scores. Moreover, for the factors, a separate data extraction tool was prepared, and it contains information on the author’s name, year of publication, and factors like driving experiences, chewing khat while driving, alcohol use, and receiving mobile phone calls while driving.

### 2.8 Statistical methods and analysis

After extracting all relevant insights into a Microsoft Excel spreadsheet, the data was then transferred to STATA software version 14 for comprehensive analysis. The combined prevalence of road traffic accidents was determined using a 95% confidence interval. Forest plots were used to show the magnitude of road traffic accidents among public transportation in Africa. Due to its help in minimizing the heterogeneity of included studies, the random effect model of analysis was used as a method of meta-analysis. Furthermore, to assess the presence of publication bias, a funnel plot was used and a more objective assessment was performed by implementing Begg and Eggers regression tests, with a significance level of *p* < 0.05 indicating the possible presence of publication bias.

Sub-group analyses were also conducted by different study characteristics such as sub-regions of Africa (Northern, South, Western, Central, and Eastern Africa), study design (cross-sectional or retrospective study), and country. Moreover, the meta-analysis regression was conducted to identify the sources of heterogeneity among studies. It was conducted using the following study-level covariates: sample size, publication year, and sub-region of included studies. The different factors associated with RTA were presented using odds ratios (OR) with a 95% confidence interval (CI). A sensitivity analysis was executed to see the effect of a single study on the overall prevalence of the meta-analysis estimate.

## 3 Results

### 3.1 Search and study selection

**Figure 1:**
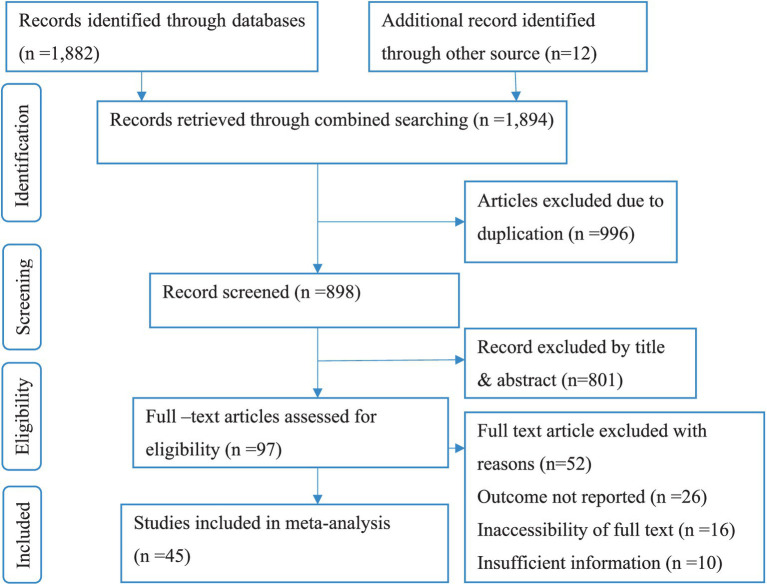
PRISMA flow diagram explaining selection of primary studies in Africa.

This systematic review included published studies conducted on the prevalence of road traffic accidents among drivers in Africa. A total of 1,894 records were retrieved through electronic database searching. From these, 996 duplicated records were excluded, and from 898 articles screened using their titles and abstracts, 801 were excluded. Ninety-seven (97), full-text articles were assessed for eligibility. From these, 52 full-text articles were excluded from *prior* criteria, and finally, 45 full-text primary articles were selected for quantitative analysis (Figure 1).

### 3.2 Characteristics of included studies

Forty- five (15–59) African countries were represented in this review with a total of 15,968 study participants. From all, 25 of the studies were from Eastern Africa (15–18, 22–24, 30–34, 39–44, 53–59), 12 were from Western African countries (12–21, 25–29, 35–38), 6 were from Northern Africa (45, 46, 48–51), one study from Southern, and one study from Central African countries (47, 52). The sample size of the included studies ranged from a minimum of 32 in a study conducted in Egypt (45) to a maximum of 862 in a study conducted in Sudan (51) of the included studies, thirty-seven were cross-sectional (15, 16, 18–20, 22–36, 38–41, 43, 44, 46, 47, 49–51, 53, 55–59), while, eight studies followed retrospective study design (17, 21, 37, 42, 45, 48, 52, 54) (Table 1).

**Table 1:** The characteristics of the studies included in the systematic review and meta-analysis.

S/N	Authors and year	Country	Sub-region	Study- design	Sample-size	Prevalence of RTA (%)	QA
1	Mekonnen et al. (15)	Ethiopia	East Africa	CS	361	16.3	9
2	Tiruneh et al. (16)	Ethiopia	East Africa	CS	830	20	9
3	Tadege (17)	Ethiopia	East Africa	RS	255	40.4	8
4	Getachew et al. (18)	Ethiopia	East Africa	CS	400	33	9
5	Poku et al. (19)	Ghana	West Africa	CS	227	55.5	9
6	Luther (20)	Ghana	West Africa	CS	387	37	9
7	Blankson et al. (21)	Ghana	West Africa	RS	860	39.1	9
8	Deresse et al. (22)	Ethiopia	East Africa	CS	554	54.7	8
9	Woldu et al. (23)	Ethiopia	East Africa	CS	246	23.8	9
10	Asefa et al. (24)	Ethiopia	East Africa	CS	712	26.4	9
11	Konlan et al. (25)	Ghana	West Africa	CS	114	64	9
12	Adejugbagbe et al. (26)	Nigeria	West Africa	CS	592	35.3	9
13	Bekibele et al. (27)	Nigeria	West Africa	CS	99	16.2	8
14	Owoaje et al. (28)	Nigeria	West Africa	CS	299	45.3	9
15	Adogu and Asuzu (29)	Nigeria	West Africa	CS	291	47.8	8
16	Boniface et al. (30)	Tanzania	East Africa	CS	675	53.4	9
17	Lwanga et al. (31)	Tanzania	East Africa	CS	290	71	8
18	Tadesse et al. (32)	Ethiopia	East Africa	CS	356	36.8	8
19	Gebresenbet et al. (33)	Ethiopia	East Africa	CS	164	36.6	9
20	Weldeslassie et al. (34)	Ethiopia	East Africa	CS	840	56.9	9
21	Okafor et al. (35)	Nigeria	West Africa	CS	315	47.9	8
22	Aliyu et al. (36)	Nigeria	West Africa	CS	300	38.3	9
23	Johnson (37)	Nigeria	West Africa	RS	200	68	9
24	Salako et al. (38)	Nigeria	West Africa	CS	300	53.7	9
25	Odiwuor et al. (39)	Kenya	East Africa	CS	166	12.1	8
26	Stanley et al. (40)	Kenya	East Africa	CS	180	38	9
27	Eric et al. (41)	Kenya	East Africa	CS	300	24	8
28	Abdulgafoor et al. (42)	Kenya	East Africa	RS	144	59.96	8
29	Tegegne et al. (43)	Ethiopia	East Africa	CS	422	23.5	9
30	Oltaye et al. (44)	Ethiopia	East Africa	CS	274	55.1	9
31	El Safty et al. (45)	Egypt	North Africa	RS	32	41.9	9
32	Badawy et al. (46)	Egypt	North Africa	CS	324	25	9
33	Nizamo et al. (47)	Mozambique	Southern	CS	282	39.4	8
34	Bodala et al. (48)	Libya	North Africa	RS	221	15.8	9
35	Elawad et al. (49)	Sudan	North Africa	CS	231	31.2	9
36	Mohammed et al. (50)	Sudan	North Africa	CS	296	33.8	9
37	Sube et al. (51)	Sudan	North Africa	CS	862	7.3	8
38	Jeannoh et al. (52)	Congo	Central	RS	134	67.9	8
39	Tumwesigye et al. (53)	Uganda	East Africa	CS	289	22	9
40	Twagirayezu et al. (54)	Rwanda	East Africa	RS	101	41.9	8
41	Patel et al. (55)	Rwanda	East Africa	CS	589	43.8	8
42	Hussen et al. (56)	Ethiopia	East Africa	CS	378	32.8	9
43	Hailemichael et al. (57)	Ethiopia	East Africa	CS	384	62.5	8
44	Hailemichael et al. (57) and Hareru et al. (58)	Ethiopia	East Africa	CS	316	39.9	9
45	Endalew et al. (59)	Ethiopia	East Africa	CS	376	17	9

CS, cross-sectional; RS, retrospective study; RTA, road traffic accidents; QA, quality assessment.

### 3.3 Pooled prevalence of RTA in Africa

A comprehensive analysis was conducted on a sample of 15,968 participants to determine the pooled prevalence of road traffic accidents (RTA) in Africa. The study included a total of twenty-five research studies in East Africa, twelve studies in West Africa, six in North Africa, one study in Southern Africa, and one study in Central African countries.

**Figure 2:**
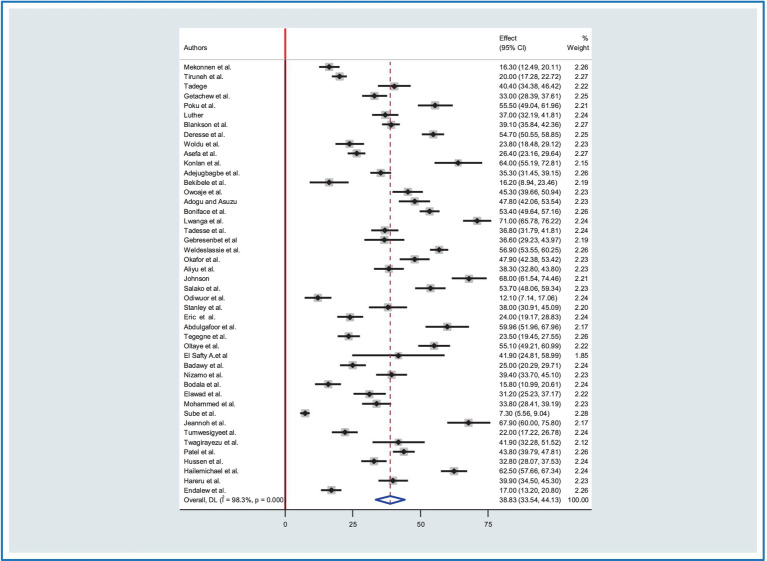
The pooled prevalence of road traffic accidents in Africa.

The overall pooled prevalence of road traffic accidents (RTA) in Africa was determined using the random-effect model with Mantel-Hanenszel heterogeneity at 38.83% (95%CI: 33.54, 44.12). This estimate was statistically significant with a *p* value of less than 0.001. Furthermore, heterogeneity between studies was found to be high with an *I*^2^ value of 98.3%, (Figure 2).

### 3.4 Sub-group meta-analysis

Before conducting a meta-analysis on the effect sizes of the studies included, it was necessary to assess the presence of statistical variability among these studies. This analysis focused on the study design, country, and sub-region. As a result, based on country, the highest pooled estimates of road traffic accidents were seen in Congo at 67.90% (95% CI: 59.99, 75.81). Whereas, the lowest pooled estimate was seen in Libya at 15.80% (95% CI: 10.99, 20.61).

On the other hand, in a sub-group analysis based on sub-region, the highest pooled estimates of road traffic accidents were seen in Central Africa at 67.90% (95% CI:59.99,75.80). Whereas, the lowest pooled estimate was seen in North Africa at 24.92% (95%CI: 13.90, 35.95). In addition, a sub-group analysis was conducted on studies using different types of study design, including cross-sectional, and retrospective study design. The prevalence of road traffic accidents in these studies was found to be 37.17% (95% CI: 31.37, 42.95), and 46.84% (95% CI: 33.79, 59.89), respectively (Table 2).

**Table 2:** Sub-group analysis of road traffic accidents in Africa.

Outcomes	Characteristics	Included studies	Total participants	Effect size (95% CI)	Heterogeneity	
					*I*^2^- value	*P*- value
RTA	Sub-region					
	West Africa	12	3,984	45.52 (39.04, 52.00)	94.4%	< 0.001
	Central Africa	1	134	67.90 (59.99, 75.80)	70.2%	<0.001
	North Africa	6	1,966	24.92 (13.90, 35.95)	97.1%	< 0.001
	East Africa	25	9,602	37.61 (31.03, 44.19)	98.1%	< 0.001
	Southern Africa	1	282	58.08 (42.03, 74.14)	98.4%	< 0.001
	Study design					
	Cross-sectional	37	14,021	37.17 (31.37, 42.95)	98.4%	< 0.001
	Retrospective study	8	1,947	46.84 (33.79, 59.89)	97.1%	< 0.001
	Country					
	Ethiopia	16	6,868	35.93 (28.17, 43.69)	98.1%	< 0.001
	Ghana	4	1,588	48.32 (37.73, 58.91)	93.7%	< 0.001
	Nigeria	8	2,396	44.09 (35.11, 53.07)	95.3%	< 0.001
	Tanzania	2	965	62.10 (44.85, 79.35)	96.5%	0.830
	Kenya	4	790	33.29 (15.16, 51.43)	71.3%	< 0.001
	Egypt	2	356	31.37 (15.32, 47.42)	97.3%	< 0.001
	Mozambique	1	282	39.40 (33.69, 45.10)	0.0%	< 0.001
	Libya	1	221	15.80 (10.99, 20.61)	0.0%	< 0.001
	Sudan	3	1,389	23.96 (40.08, 43.84)	98.5%	< 0.001
	Congo	1	134	67.90 (59.99, 75.81)	0.0%	< 0.001
	Uganda	1	289	22.00 (17.22, 26.77)	0.0%	< 0.001
	Rwanda	2	690	43.52 (39.82, 47.22)	96.3%	< 0.001
Total	45		15,968	38.83 (33.54, 44.12)	98.3%	< 0.001

### 3.5 Sensitivity meta-analyses

To examine the possible influence of individual studies on the overall pooled prevalence effect, a leave-one-out sensitivity analysis was performed. The results showed that no specific study had a significant impact on the overall pooled prevalence of road traffic accidents in Africa. The results showed that the combined effect remained significantly unchanged even after excluding a specific study (Table 3).

**Table 3:** Sensitivity analysis of pooled prevalence with each study was removed one by one.

Study omitted	Estimate	95% Conf. interval	
Study of RTA			
Mekonnen et al. (15)	39.35	33.98	44.72
Tiruneh et al. (16)	39.27	33.83	44.71
Tadege (17)	38.80	33.42	44.18
Getachew et al. (18)	38.97	33.55	44.38
Poku et al. (19)	38.45	33.12	43.78
Luther (20)	38.87	33.47	44.28
Blankson et al. (21)	38.83	33.37	44.28
Deresse et al. (22)	38.46	33.15	43.77
Woldu et al. (23)	39.17	33.79	44.56
Asefa et al. (24)	39.12	33.66	44.58
Konlan et al. (25)	38.28	32.96	43.59
Adejugbagbe et al. (26)	38.91	33.48	44.35
Bekibele et al. (27)	39.34	33.98	44.69
Owoaje et al. (28)	38.68	33.31	44.06
Adogu and Asuzu (29)	38.63	33.26	43.99
Boniface et al. (30)	38.49	33.18	43.81
Lwanga et al. (31)	38.09	32.91	43.26
Tadesse et al. (32)	38.88	33.48	44.28
Gebresenbet et al. (33)	38.88	33.51	44.25
Weldeslassie et al. (34)	38.41	33.17	43.65
Okafor et al. (35)	38.62	33.26	43.99
Aliyu et al. (36)	38.84	33.45	44.23
Johnson (37)	38.17	32.91	43.43
Salako et al. (38)	38.49	33.15	43.83
Odiwuor et al. (39)	39.44	34.10	44.78
Stanley et al. (40)	38.85	33.48	44.22
Eric et al. (41)	39.17	33.78	44.57
Abdulgafoor et al. (42)	38.36	33.04	43.68
Tegegne et al. (43)	39.19	33.77	44.60
Oltaye et al. (44)	38.46	33.13	43.79
El Safty et al. (45)	38.77	33.43	44.12
Badawy et al. (46)	39.15	33.75	44.55
Nizamo et al. (47)	38.82	33.43	44.20
Bodala et al. (48)	39.36	34.00	44.72
Elawad et al. (49)	39.00	33.62	44.39
Mohammed et al. (50)	38.95	33.55	44.34
Sube et al. (51)	39.54	34.91	44.17
Jeannoh et al. (52)	38.18	32.89	43.47
Tumwesigye et al. (53)	39.22	33.83	44.61
Twagirayezu et al. (54)	38.76	33.40	44.12
Patel et al. (55)	38.72	33.32	44.12
Hussen et al. (56)	38.97	33.56	44.38
Hailemichael et al. (57)	38.28	33.03	43.53
Hareru et al. (58)	38.81	33.42	44.20
Endalew et al. (59)	39.34	33.96	44.71
Combined	38.83	33.543	44.12

### 3.6 Meta-regression

Meta-regression, along with sub-group and sensitivity analysis, was used to identify sources of heterogeneity, but no significant variables were found. Therefore, heterogeneity can be explained by other factors not included in this review (Table 4).

**Table 4:** Meta-regression analysis of factors affecting between-study heterogeneity.

Source of heterogeneity	Coefficient	Standard error	*P*-value
Year of publication	0.94	0.07	0.46
Country	0.92	0.10	0.54
Sample size	0.99	0.01	0.40
Study design	1.47	1.64	0.72
Sub-region	1.05	0.31	0.85

### 3.7 Publication bias

**Figure 3:**
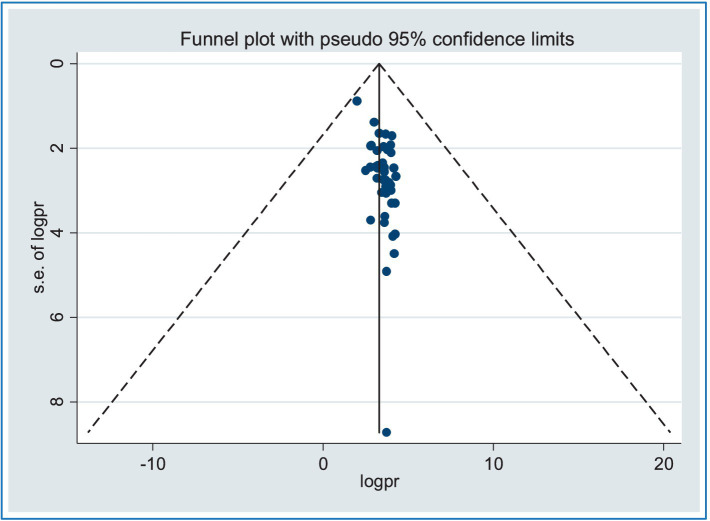
Funnel plots for publication bias of road traffic accidents in Africa.

The presence of publication bias was assessed using a funnel plot and the application of the Egger’s and Begg’s regression test at a significant level of 5%. The symmetrical arrangement of the included studies, as shown in the funnel plot, indicated the absence of publication bias. Furthermore, there was no statistical evidence to support the existence of publication bias, and Begg’s and Egger’s tests yielded *p*-values of 0.43 and 0.23, respectively, which were not statistically significant. Consequently, the test results provide no evidence of a small study effect (Figure 3).

### 3.8 Factors associated with RTA in Africa

We performed a meta-analysis to identify associated factors for road traffic accidents using the random effects model. During the extraction process, we planned to show the association of each factor with the outcome variable. Therefore, we examined the pooled effect of four factors on the outcome variable such as driving experience, chewing Khat while driving, alcohol use, and receiving mobile Phone calls while driving.

**Figure 4:**
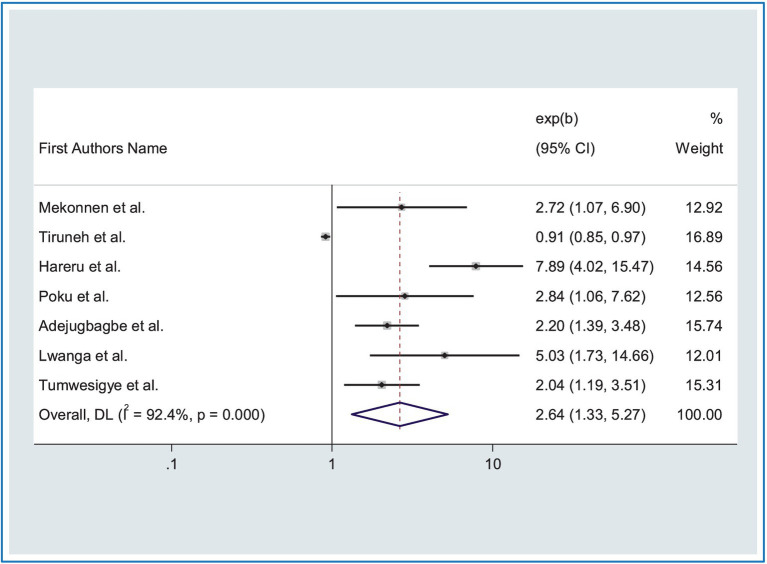
Forest plot showing the association between RTA and driving experiences.

In this meta-analysis, factors associated with road traffic accidents (RTA) were assessed using thirteen studies (15, 16, 18, 19, 23, 24, 26, 31, 32, 41, 53, 56, 58). Among thirteen articles, the findings of seven studies (15, 16, 19, 26, 31, 53, 58) revealed that road traffic accidents were significantly associated with driving experiences. As a result, the probability of road traffic accidents was 2.64 times greater for public transportation lacking driving experience compared to those who possess such experience (AOR: 2.64, 95% CI:1.70, 3.34) (Figure 4). Higher heterogeneity was observed across studies (*I*^2^ = 92.40%, *p* = 0.00), for this reason, we used a random effects model.

**Figure 5:**
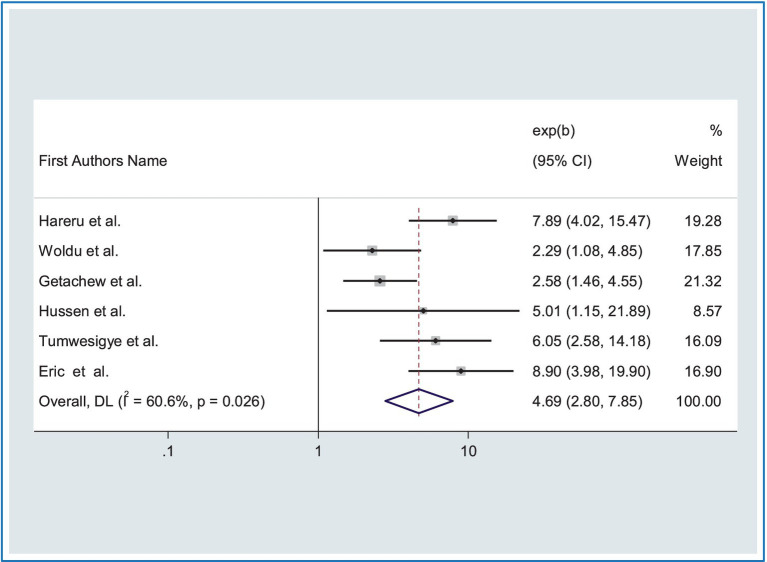
Forest plot showing the association between RTA and Chewing Khat while driving.

The relationship between road traffic accidents and chewing Khat while driving has been assessed in six studies (18, 23, 41, 53, 56, 58). Drivers who chewed Khat while driving were 4.69 times (AOR = 4.69, CI: 2.80,7.85) more likely to be at risk of road traffic accidents as compared to taxi drivers who did not chew Khat while driving (Figure 5). Moderate heterogeneity was observed across studies (*I*^2^ = 60.60%, *p* = 0.02), for this reason, we used a random effects model.

**Figure 6:**
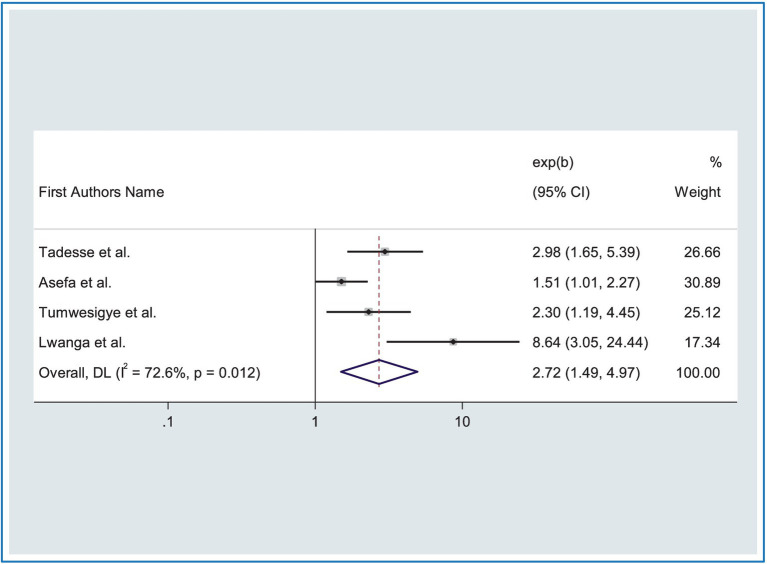
Forest plot showing the association between RTA and alcohol use.

The relationship between road traffic accidents and alcohol use has been assessed in four studies (24, 31, 32, 53). The result showed that the combined effect of alcohol use was significantly associated with RTA. Drivers who used alcohol while driving were 2.72 times (AOR = 2.72, CI: 1.49, 4.97) more likely to be at risk of road traffic accidents (RTA) as compared to taxi drivers who did not use alcohol while driving (Figure 6). Moderate heterogeneity was observed across studies (*I*^2^ = 72.60%, *p* = 0.01), for this reason, we used a random effects model.

**Figure 7:**
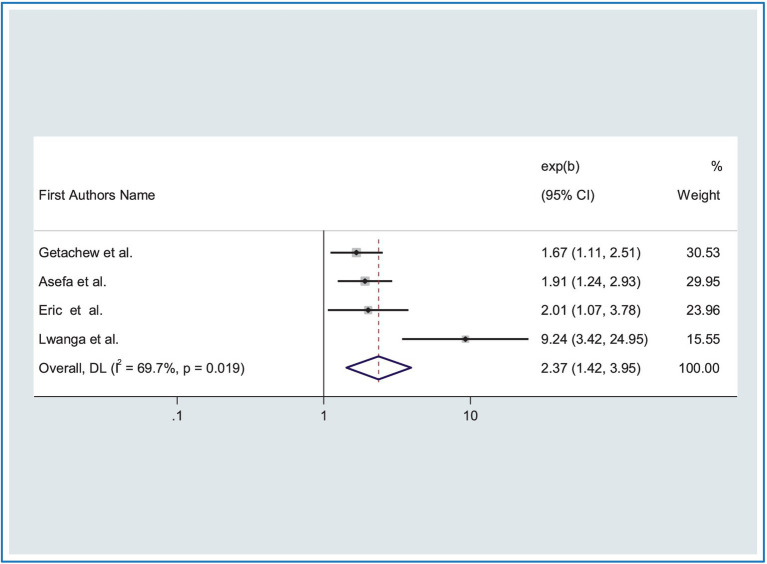
Forest plot showing the association between RTA and receiving mobile phone calls while driving.

Finally, the four studies showed that road traffic accidents were significantly associated with receiving mobile Phone calls while driving (18, 24, 31, 41). Drivers who received mobile Phone calls while driving were 2.37 times (AOR = 2.37, CI: 1.42, 3.95) more likely to be at risk of road traffic accidents (RTA) as compared to taxi drivers who did not receive mobile Phone calls while driving. Because heterogeneity was moderate, we used a random effects model (*I*^2^ = 69.7%, *p* = 0.01) (Figure 7).

## 4 Discussion

This meta-analysis study was the first study that assessed the pooled prevalence of RTA and their associated factors in Africa. A total of 45 studies were included in the review. The overall pooled prevalence of road traffic accidents in Africa was found to be 38.83%. This finding is higher than a study conducted in Iran (16.4%), India (35.2%), and Brazil (20.8%) (60–62). The discrepancy may be due to infrastructure variations, like traffic signals at intersections, which help ensure driver compliance with regulations and reduce accident likelihood. Moreover, the interplay between socio-economic conditions and infrastructure development plays a critical role in shaping the quality of roads, ultimately impacting overall road safety and efficiency.

In this study, the sub-group analysis result by country showed that the higher pooled prevalence of road traffic accidents was found in Congo at 67.90%, There was an increment of accidents in this study compared with the previous study done in India, which showed that the prevalence of road traffic accident was 35.2% (61), and another study in India, on prevalence of road traffic accident showed that the prevalence was 31% (63). This dissimilarity might be due to variations in the study setting and implementation of driving rules and regulations.

Moreover, the pooled prevalence of road traffic accident increment was seen in Central Africa at 67.90%, after sub-group analysis was performed based on sub-region. This finding is higher than a study conducted in India (35.2%), and Brazil (20.8%) (61, 62). The primary distinctions between contemporary research and earlier investigations into road traffic accidents can be ascribed to insufficient safety awareness and the endorsement of drivers who lacked proper training.

In this study, road traffic accidents were significantly associated with driving experiences. As a result, the probability of road traffic accidents was 2.64 times greater for public transportation lacking driving experiences compared to those who possess such experiences. This finding is supported by the study conducted in Iran (60), China (64), and Australia (65). The phenomenon may arise from expectations of dangerous traffic conditions requiring extensive practice. Novice drivers often exceed speed limits, overestimate their abilities, and underestimate risks. This indicates that a solid understanding of risk and commitment to safe driving develops with years of experience.

Furthermore, in the present study, drivers who chewed Khat while driving were 4.69 times more likely to be at risk of road traffic accidents as compared to taxi drivers who did not chew Khat while driving. This finding was supported by a study conducted in Australia (65), and Iran (60). A possible explanation for this observation may relate to the economic conditions of taxi drivers, the composition of road users, traffic patterns or density, the legal framework, and various socio-cultural factors that could influence the perception or reality of rapid driving as a contributing factor to road traffic accidents across different countries and contexts.

Moreover, this study revealed that driving after taking alcohol was found to be an aggravating factor for road traffic accidents. Drivers who drove after consuming alcohol were 2.72 more likely to have road traffic accidents compared with those who did not consume alcohol. This finding was supported by a study conducted in Australia (65), and China (64). This might be due to the nature of alcohol which has a range of psychomotor and cognitive effects, including attitude, judgment, vigilance, perception, reaction, and control (66). This can increase accident risk by lowering cognitive processing, coordination, attention, vision, and hearing.

Furthermore, the likelihood of road traffic accidents was 2.37 times higher among drivers who used cell phones while driving compared with those who did not use them. This study is consistent with previously done studies that have also reported that drivers distracted by mobile devices such as smartphones and/or other in-vehicle devices are at risk for serious negative outcomes (67). This is because of a loss of attention to surroundings while driving.

The research emphasizes the need for governments to proactively address road traffic accidents through comprehensive strategy, focusing on strict enforcement of traffic laws, especially against common violations like speeding and ignoring signals. Governments ought to invest in extensive driver education and training programs to improve awareness of road safety and promote responsible driving behaviors.

Policies must prioritize coordinated actions to reduce substance abuse among drivers through strict enforcement of regulations on alcohol and drug impairment. Moreover, Public awareness initiatives regarding the hazards associated with substance use and the reception of mobile phone calls while operating a vehicle could significantly contribute to the reduction of these risks. By implementing such strategies, African nations have the potential to foster a culture of road safety awareness, which may ultimately lead to a decrease in the incidence of road traffic accidents.

## 5 Limitations of the study

This research represents a pioneering systematic review and meta-analysis aimed at estimating the aggregated prevalence and associated risk factors of road traffic accidents across Africa. Nonetheless, the lack of studies from nations outside those included may restrict the overall representativeness of the findings for the continent. In addition, emphasis on English-language publications in the study introduces a potential language bias, thereby constraining the range of research examined. Although Egger’s test was employed to identify publication bias, its validity may be undermined by the small number of studies analyzed, as those yielding positive results are often more likely to be published. The existence of unexamined heterogeneity, despite the execution of subgroup analyses, indicates that there may be other factors affecting the outcomes that have not been considered.

## 6 Conclusion

In conclusion, the pooled prevalence of RTA in Africa was high. Because of different factors like driving experience, chewing Khat while driving, alcohol consumption, and receiving mobile phone calls while driving. Therefore, the authors recommend that drivers have to strictly adhere to traffic regulations. Pedestrians must follow traffic rules and exercise caution when crossing roads.

## Data Availability

The original contributions presented in the study are included in the article/Supplementary material, further inquiries can be directed to the corresponding author.
