# Supplementary material for: Epidemiology of road traffic accidents and its associated factors among public transportation in Africa: systematic review and meta-analysis
Source: Front Public Health. 2025 Feb 28;13:1511715. doi: 10.3389/fpubh.2025.1511715 (PMC11908462; doi:10.3389/fpubh.2025.1511715)
Supplement: Supplementary file 2 [file Table_2.docx]

**S2-Table**. Quality assessment for the included Studies

| **Item** | **Clearly defined incluion criteria** | **Describe the study setting and participant** | **Valid and reliable exposure measurement** | **Objective and standard criteria for measurement** | **Identified confounder** | **Strategies to deal with confounders** | **Valid and reliable outcome measurement** | **Appropriate static analysis** | **No of ‘yes’ ‘** |
| --- | --- | --- | --- | --- | --- | --- | --- | --- | --- |
| Mekonnen et al. | Yes | Yes | No | Yes | Yes | No | Yes | Yes | 6/8=75 |
| Tiruneh et al. | Yes | Yes | Yes | Yes | No | No | Yes | Yes | 6/8=75 |
| Tadege | Yes | Yes | No | Yes | Yes | No | Yes | Yes | 6/8=75 |
| Getachew et al. | Yes | Yes | No | Yes | Yes | Yes | Yes | Yes | 7/8=87.5 |
| Poku et al. | Yes | Yes | No | Yes | Yes | Yes | Yes | Yes | 7/8=87.5 |
| Luther | Yes | Yes | Yes | Yes | Yes | No | Yes | Yes | 7/8=87.5 |
| Blankson et al. | Yes | Yes | No | Yes | Yes | Yes | Yes | Yes | 7/8=87.5 |
| Deresse et al. | Yes | Yes | Yes | Yes | No | No | Yes | Yes | 6/8=75 |
| Woldu et al. | Yes | Yes | No | Yes | Yes | Yes | Yes | No | 6/8=75 |
| Asefa et al. | Yes | Yes | Yes | Yes | No | Yes | Yes | No | 6/8=75 |
| Konlan et al. | Yes | Yes | Yes | Yes | No | No | Yes | Yes | 6/8=75 |
| Adejugbagbe et al. | Yes | yes | No | Yes | Yes | Yes | Yes | Yes | 7/8=87.5 |
| Bekibele et al. | Yes | Yes | Yes | No | Yes | No | Yes | Yes | 6/8=75 |
| Owoaje et al. | Yes | Yes | Yes | Yes | Yes | Yes | Yes | No | 7/8=87.5 |
| Adogu and Asuzu | Yes | Yes | Yes | Yes | Yes | Yes | No | Yes | 7/8=87.5 |
| Boniface et al. | Yes | Yes | No | Yes | Yes | Yes | Yes | Yes | 7/8=87.5 |
| Lwanga et al. | Yes | Yes | No | Yes | No | Yes | Yes | Yes | 6/8=75 |
| Tadesse et al. | Yes | Yes | Yes | Yes | Yes | Yes | Yes | No | 7 /8=87.5 |
| Gebresenbet et al. | Yes | Yes | No | Yes | Yes | No | Yes | Yes | 6/8=75 |
| Weldeslassie et al. | Yes | Yes | Yes | Yes | Yes | Yes | Yes | No | 7/8=87.5 |
| Okafor et al. | Yes | Yes | Yes | No | Yes | Yes | Yes | No | 6 / 8=75 |
| Aliyu et al. | Yes | Yes | No | Yes | Yes | Yes | Yes | Yes | 7/8=87.5 |
| Johnson | Yes | Yes | No | Yes | Yes | Yes | Yes | Yes | 7/8=87.5 |
| Salako et al. | Yes | Yes | No | Yes | Yes | Yes | Yes | Yes | 7/8=87.5 |
| Odiwuor et al. | Yes | Yes | Yes | Yes | Yes | Yes | Yes | No | 7/ 8=87.5 |
| Stanley et al. | Yes | Yes | Yes | No | Yes | Yes | No | Yes | 6/8=75 |
| Eric et al. | Yes | Yes | Yes | No | Yes | Yes | Yes | Yes | 7/8=87.5 |
| Abdulgafoor et al. | Yes | Yes | No | Yes | Yes | Yes | Yes | No | 6/8=75 |
| Tegegne et al. | Yes | Yes | Yes | Yes | Yes | Yes | Yes | No | 7/8=75.5 |
| Oltaye et al. | Yes | Yes | No | Yes | Yes | Yes | No | Yes | 7/8=75.5 |
| El Safty A.et al. | Yes | Yes | Yes | Yes | Yes | No | Yes | Yes | 7/8=75.5 |
| Badawy et al. | Yes | Yes | Yes | Yes | No | Yes | Yes | No | 6/8=75 |
| Nizamo et al. | Yes | Yes | No | Yes | Yes | Yes | No | Yes | 6/8=75 |
| Bodala et al. | Yes | Yes | Yes | Yes | Yes | Yes | No | Yes | 7/8=75.5 |
| Elawad et al. | Yes | Yes | No | Yes | Yes | Yes | Yes | Yes | 7/8=75.5 |
| Mohammed et al. | Yes | Yes | No | Yes | Yes | No | Yes | Yes | 6/8=75 |
| Sube et al. | Yes | Yes | No | Yes | Yes | No | Yes | Yes | 6/8=75 |
| Jeannoh et al. | Yes | Yes | Yes | Yes | Yes | Yes | No | Yes | 7/8=75.5 |
| Tumwesigyeet al. | Yes | Yes | Yes | Yes | Yes | Yes | No | Yes | 7/8=75.5 |
| Twagirayezu et al. | Yes | Yes | Yes | Yes | No | Yes | No | Yes | 6/8=75 |
| Patel et al. | Yes | Yes | Yes | No | Yes | Yes | Yes | Yes | 7/8=75.5 |
| Hussen et al. | Yes | Yes | No | Yes | Yes | No | Yes | Yes | 6/8=75 |
| Hailemichael et al. | Yes | Yes | No | Yes | Yes | No | Yes | Yes | 6/8=75 |
| Hareru et al. | Yes | Yes | No | Yes | Yes | No | Yes | Yes | 6/8=75 |
| Endalew et al. | Yes | Yes | No | Yes | Yes | No | Yes | Yes | 6/8=75 |
