# Supplementary material for: Epidemiology of road traffic accidents and its associated factors among public transportation in Africa: systematic review and meta-analysis
Source: Front Public Health. 2025 Feb 28;13:1511715. doi: 10.3389/fpubh.2025.1511715 (PMC11908462; doi:10.3389/fpubh.2025.1511715)
Supplement: Supplementary file 3 [file Table_3.docx]

**S3-Table**. Risk of bias assessment for the included studies

| **Item** | **External validity** | | | | **Internal validity** | | | | | |  | |
| --- | --- | --- | --- | --- | --- | --- | --- | --- | --- | --- | --- | --- |
|  | Representativeness of the target population | Representativeness of the sampling frame | Radom sampling or census | Minimal response bias | Data were collected directly | Acceptable case definition used in the study | Valid and reliable measurement | The same mode of data collection for all study subject | Appropriate length of prevalence period for parameter of interest | Appropriate numerators and denominator s of interest | No of yes | Sum ery of risk of bias |
| Mekonnen et al. | Yes | Yes | Yes | Yes | No | No | Yes | Yes | Yes | Yes | 9 | Low- risk |
| Tiruneh et al. | Yes | Yes | No | Yes | Yes | No | Yes | Yes | Yes | Yes | 9 | Low- risk |
| Tadege | Yes | Yes | No | Yes | Yes | Yes | Yes | Yes | Yes | Yes | 8 | Low – risk |
| Getachew et al. | Yes | Yes | No | Yes | Yes | Yes | Yes | Yes | Yes | Yes | 9 | Low- risk |
| Poku et al. | Yes | Yes | Yes | Yes | Yes | No | Yes | Yes | Yes | Yes | 9 | Low- risk |
| Luther | Yes | Yes | No | Yes | Yes | Yes | Yes | Yes | Yes | Yes | 9 | Low- risk |
| Blankson et al. | Yes | Yes | Yes | Yes | No | No | Yes | Yes | Yes | Yes | 9 | Low- risk |
| Deresse et al. | Yes | Yes | No | Yes | Yes | Yes | Yes | No | Yes | Yes | 8 | Low- risk |

| Woldu et al. | Yes | Yes | Yes | Yes | No | Yes | Yes | No | Yes | Yes | 9 | Low- risk |
| --- | --- | --- | --- | --- | --- | --- | --- | --- | --- | --- | --- | --- |
| Asefa et al. | Yes | Yes | Yes | Yes | No | No | Yes | Yes | Yes | Yes | 9 | Low-risk |
| Konlan et al. | Yes | yes | No | Yes | Yes | Yes | Yes | Yes | Yes | Yes | 9 | Low- risk |
| Adejugbagbe et al. | Yes | Yes | Yes | No | Yes | No | Yes | Yes | Yes | Yes | 9 | Low-risk |
| Bekibele et al. | Yes | Yes | Yes | Yes | Yes | Yes | Yes | No | Yes | Yes | 8 | Low-risk |
| Owoaje et al. | Yes | Yes | Yes | Yes | Yes | Yes | No | Yes | Yes | Yes | 9 | Low- risk |
| Adogu and Asuzu | Yes | Yes | No | Yes | Yes | Yes | Yes | Yes | Yes | Yes | 8 | Low risk |
| Boniface et al. | Yes | Yes | Yes | Yes | Yes | Yes | Yes | Yes | No | No | 9 | Low- risk |
| Lwanga et al. | Yes | Yes | Yes | Yes | Yes | Yes | Yes | No | No | Yes | 8 | Low- risk |
| Tadesse et al. | Yes | Yes | Yes | Yes | Yes | Yes | Yes | Yes | Yes | No | 8 | Low- risk |
| Gebresenbet et al. | Yes | Yes | Yes | Yes | Yes | Yes | Yes | No | No | Yes | 9 | Low -risk |
| Weldeslassie et al. | Yes | Yes | Yes | No | Yes | Yes | Yes | No | Yes | Yes | 9 | Low -risk |
| Okafor et al. | Yes | Yes | Yes | No | Yes | Yes | Yes | Yes | Yes | Yes | 8 | Low-risk |
| Aliyu et al. | Yes | Yes | Yes | Yes | Yes | Yes | Yes | No | Yes | No | 9 | Low-risk |
| Johnson | Yes | Yes | Yes | No | No | Yes | Yes | Yes | Yes | Yes | 9 | Low-risk |
| Salako et al. | Yes | Yes | Yes | Yes | No | Yes | Yes | Yes | Yes | Yes | 9 | Low-risk |
| Odiwuor et al. | Yes | No | Yes | No | Yes | Yes | Yes | Yes | Yes | Yes | 8 | Low-risk |
| Stanley et al. | Yes | Yes | Yes | Yes | Yes | Yes | Yes | No | Yes | Yes | 9 | Low-risk |
| Eric et al. | Yes | Yes | Yes | Yes | No | Yes | Yes | Yes | No | Yes | 8 | Low-risk |
| Abdulgafoor et al. | Yes | Yes | No | Yes | Yes | Yes | Yes | Yes | Yes | Yes | 8 | Low-risk |
| Tegegne et al. | Yes | Yes | Yes | Yes | Yes | No | Yes | Yes | Yes | Yes | 9 | Low-risk |
| Oltaye et al. | Yes | Yes | Yes | Yes | Yes | Yes | No | Yes | Yes | Yes | 9 | Low-risk |
| El Safty A.et al. | Yes | Yes | Yes | Yes | Yes | Yes | Yes | No | Yes | No | 9 | Low-risk |
| Badawy et al. | Yes | No | Yes | Yes | No | Yes | Yes | Yes | Yes | Yes | 9 | Low-risk |
| Nizamo et al. | Yes | Yes | Yes | Yes | Yes | Yes | Yes | Yes | No | Yes | 8 | Low-risk |
| Bodala et al. | Yes | Yes | Yes | No | Yes | No | Yes | Yes | Yes | Yes | 9 | Low-risk |
| Elawad et al. | Yes | Yes | Yes | Yes | Yes | Yes | No | Yes | Yes | Yes | 9 | Low-risk |
| Mohammed et al. | Yes | Yes | No | Yes | Yes | Yes | Yes | No | Yes | Yes | 9 | Low-risk |
| Sube et al. | Yes | Yes | Yes | No | Yes | Yes | Yes | Yes | Yes | Yes | 8 | Low-risk |
| Jeannoh et al. | Yes | Yes | Yes | Yes | Yes | Yes | Yes | No | Yes | Yes | 8 | Low-risk |
| Tumwesigyeet al. | Yes | Yes | Yes | Yes | No | Yes | Yes | Yes | No | Yes | 9 | Low-risk |
| Twagirayezu et al. | Yes | Yes | Yes | Yes | Yes | No | Yes | Yes | Yes | Yes | 8 | Low-risk |
| Patel et al. | Yes | Yes | Yes | Yes | Yes | Yes | Yes | No | Yes | Yes | 8 | Low-risk |
| Hussen et al. | Yes | Yes | Yes | Yes | No | Yes | Yes | Yes | No | Yes | 9 | Low-risk |
| Hailemichael et al. | Yes | Yes | Yes | Yes | Yes | Yes | Yes | No | Yes | Yes | 8 | Low-risk |
| Hareru et al. | Yes | Yes | Yes | Yes | Yes | Yes | Yes | No | No | Yes | 9 | Low-risk |
| Endalew et al. | Yes | Yes | Yes | Yes | Yes | Yes | Yes | No | Yes | Yes | 9 | Low-risk |
